# Toward a digital decision- and workflow-support system for initiation and control of long-term non-invasive ventilation in stable hypercapnic COPD patients

**DOI:** 10.1177/20406223221099338

**Published:** 2022-05-25

**Authors:** Christian Gabriel Cornelissen, Stefan Winter, Daniel Keuchel, Nicolai Spicher, Britta Boeckmann, Christian Stephan, Tan Saygi, Wolfram Windisch, Thomas Vollmer, Michael Dreher

**Affiliations:** Department of Pulmonology and Intensive Care Medicine, University Hospital Aachen, 52074 Aachen, Germany; Philips GmbH Innovative Technologies Aachen, Aachen, Germany; Fachhochschule Dortmund, FB Informatik, Darmstadt, Germany; Fachhochschule Dortmund, FB Informatik, Darmstadt, Germany; Fachhochschule Dortmund, FB Informatik, Darmstadt, Germany; Kairos GmbH, Bochum, Germany; Kairos GmbH, Bochum, Germany; Department of Pulmonology, Cologne Merheim Hospital, Kliniken Der Stadt Köln gGmbH, Witten/Herdecke University, Cologne, Germany; Philips GmbH Innovative Technologies Aachen, Aachen, Germany; Department of Pulmonology and Intensive Care Medicine, University Hospital Aachen, Aachen, Germany

**Keywords:** COPD, decision-support system, hypercapnia, NIV, non-invasive ventilation

## Abstract

**Introduction:**

Due to an increasing demand for the initiation and control of non-invasive ventilation (NIV), digital algorithms are suggested to support therapeutic decisions and workflows in an ambulatory setting. The DIGIVENT project established and implemented such algorithms for patients with chronic hypercapnic respiratory failure due to chronic obstructive pulmonary disease (COPD) by a predefined process.

**Methods:**

Based on long-term clinical experience and guideline recommendations as provided by the German Respiratory Society, detailed graphical descriptions of how to perform NIV in stable COPD patients were created. Subsequently, these clinical workflows were implemented in the Business Process Model and Notation (BPMN) as one tool to formalize these workflows serving as input for an executable digital implementation.

**Results:**

We succeeded in creating an executable digital implementation that reflects clinical decision-making and workflows in digital algorithms. Furthermore, we built a user-friendly graphical interface that allows easy interaction with the DIGIVENT support algorithms.

**Conclusion:**

The DIGIVENT project established digital treatment algorithms and implemented a decision- and workflow-support system for NIV whose validation in a clinical cohort is planned.

## Introduction

The demand for initiation and control of non-invasive ventilation (NIV) is steadily increasing and remains resource-intensive even in an ambulatory setting. It also requires a highly specialized medical team. Digital algorithms are suggested to support therapeutic decisions and workflows in an ambulatory setting ensuring that the process of NIV initiation and control is accessible also for less-specialized staff.

The DIGIVENT project established and implemented such algorithms for patients with chronic hypercapnic respiratory failure due to chronic obstructive pulmonary disease (COPD) by a predefined process. Thereby, experienced respiratory physicians created detailed graphical descriptions of the current clinical practice and the available evidence on how to perform NIV in stable COPD patients.^1–7^ These descriptions serve as input for establishing a computer executable graphical representation of the clinical workflows in the Business Process Model and Notation (BPMN) as one tool to formalize these workflows. The BPMN representation allows an executable digital implementation of these workflows, reflecting clinical decision-making and workflows in digital algorithms. A user-friendly graphical interface allows easy interaction with the DIGIVENT support algorithms.

The aim of this communication is to describe in detail the process of creating digital algorithms, which are dedicated to support initiation and control of NIV in stable hypercapnic COPD patients. The authors are planning a clinical study to evaluate the processes described in this manuscript.

## Description and analysis of current clinical practice

Two experienced respiratory physicians from the Department of Pulmonology and Intensive Care Medicine at the University Hospital Aachen, Germany, composed the processes of initiating and controlling NIV in written graphical form in detail. This was based on long-term clinical experience and guideline recommendations as provided by the German Respiratory Society^6,7^ (S2-LL). The descriptions were redacted internally by a second group of two experienced respiratory physicians before an external group of respiratory physicians performed an additional review of the descriptions.

## Formalized processes

Three blocks of processes were identified that suffice to describe the current clinical practice:

1. First adaption of NIV (INI).
2. Titration of NIV during day-time (TDT) – (a) for patients who start NIV (after the initiation process); (2) for patients presenting for control visit of NIV after a first night of ventilation; (3) for patients who start NIV after night time ventilation needing further adjustments of NIV.
3. Recommendation for adjustments/titration or against adjustments (if nocturnal NIV is sufficient) after night time NIV (TNT).

**Figure 1.**
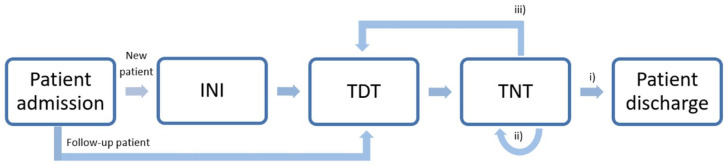
Structure of the decision-support processes: every new patient undergoes the INI-process (first initiation of NIV) before TDT (titration of NIV during daytime) and TNT (titration of NIV during night time) processes are applied. If the patient is recurring, the TNT process is performed. Within the TNT process, the algorithm indicates whether the patient is discharged (option a), repeats the TNT process (option b), or repeats both the TDT and TNT process (option c).

The relation of the three blocks is depicted in Figure 1. Each process has predefined aims regarding the quality of ventilation and patient satisfaction that the algorithms seeks to achieve. The first category is assessed by peripheral oxygen saturation (SpO_2_), pH, and PaCO_2_, and the latter is assessed by a patient questionnaire:

Q1. How much air do you get during ventilation? [enough/too much/too little] – reflecting inspiratory pressure.
Q2. How fast does the ventilator deliver the air? [adequately/too fast/too slow] – reflecting rise time.
Q3. Is it exhausting to initiate a new breath? [yes/no] – reflecting trigger sensitivity.
Q4. Is the ventilation now more comfortable than in the previous configuration? [yes/no].

### INI: first initiation of NIV

The process for first initiation of NIV (INI) aims for applying a ‘best-practice’ ventilation to the patients, followed by an optimization of inspiratory trigger sensitivity, inspiratory pressure, and rise time. The respirator is set to an initial configuration feasible for most patients (Mode: pressure support ventilation; IPAP: 16 mbar; EPAP 5 mbar; backup frequency 18/minute). A suitable mask is chosen by a physician, and the patient is ventilated using this configuration for 5 minutes. Depending on the answers to the questionnaire (Q1, Q2, and Q3), the algorithm recommends adjustments to the configuration of the respirator to reach an inspiratory pressure of up to 20 mbar – however, regarding patients’ perception inspiratory, pressure can be lower than 20 mbar.

Following termination of the process for INI, the patient undergoes the process for titration of NIV during daytime (TDT).

### TDT: titration of NIV during day-time

**Figure 2.**
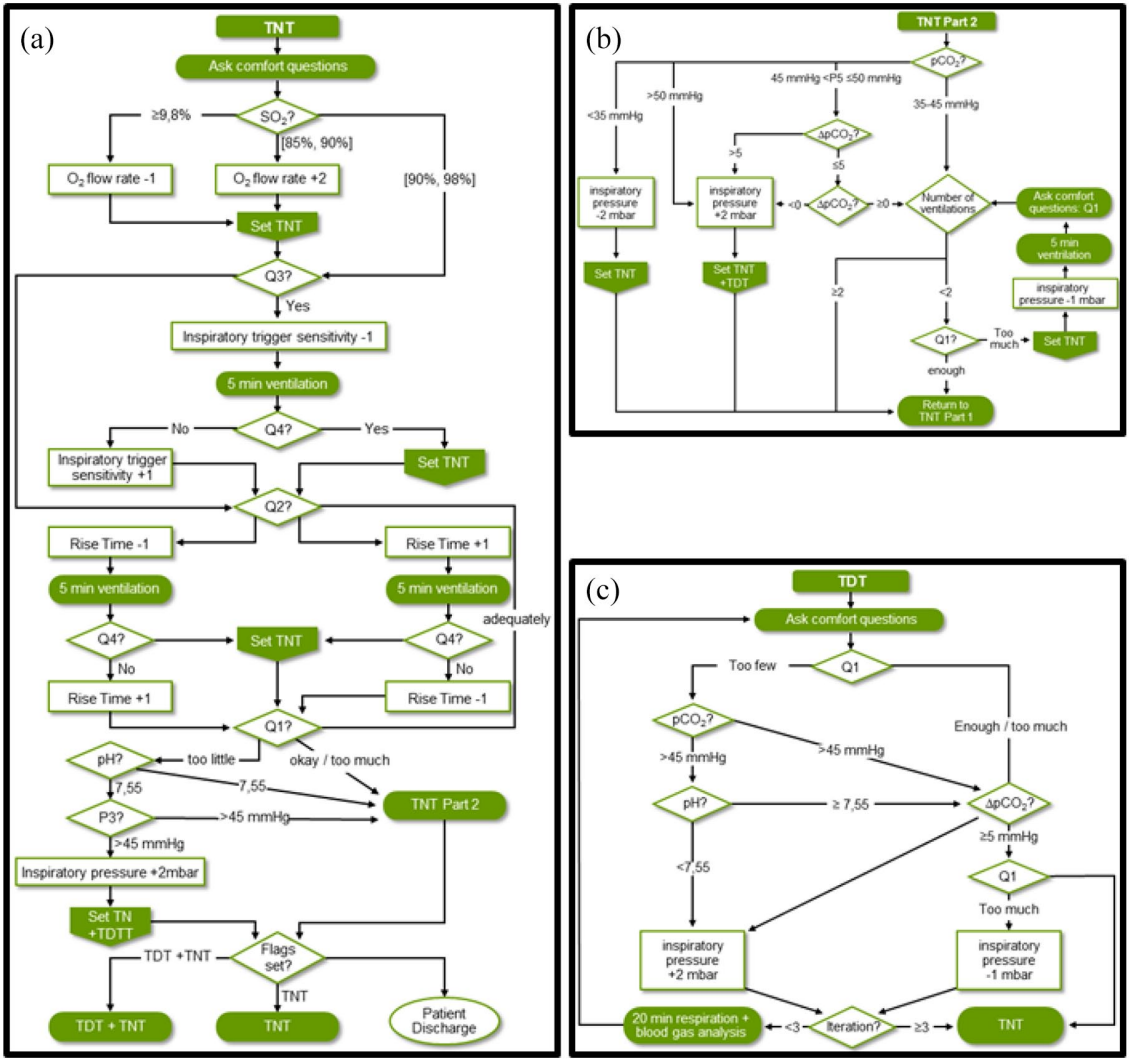
Night time (TNT) and day-time (TDT) titration processes: rectangles represent changes in the configuration of the respirator, diamonds represent decisions, and rectangles with rounded corners are performed activities. A badge symbol is used which defines planned activities for the patient (‘flags’) which are evaluated at the end of the process: (a) Part 1 of TNT process and (b) Part 2 of TNT process. If PaCO_2_ is in the interval (45–50) mmHg, the algorithm evaluates whether the current value is improved more than 5 mmHg compared to the lowest value of this patient and if the current value is improved compared to the previous measured value. (c) TDT process for optimizing inspiratory pressure.

The process for TDT has the aim to set the inspiratory pressure to an optimal value. Regarding follow-up patients, the ventilator is set with the configuration from in-hospital night time settings. Regarding patient initiation, the configuration of the ventilator from the process for INI or from consecutive night time ventilation is kept. Subsequently, the patient is ventilated for 20 minutes. After 15 minutes, a blood gas analysis is performed and evaluated:

1. If hypoxemia (SpO_2_ < 85%) or acidosis (pH < 7.35) occurs, the process is aborted immediately and the algorithm advises to call a physician.
2. If alkalosis occurs (pH > 7.55), the system recommends stopping ventilation and the process is paused for 30 minutes. Subsequently, a blood gas analysis is performed. If the condition disappeared (pH ⩽ 7.55) and the patient approves, the process is initiated again with the IPAP lowered by 2 mbar, starting with the step of 20 minutes ventilation. Otherwise, the process is aborted.
3. If the values are within safety ranges, the patient is asked the patient satisfaction question Q1 and recommendations on how to optimize the inspiratory pressure are given as shown in Figure 2(a).

This cycle of ventilation and parameter change is repeated up to three times if the patient approves. Following, the process for TDT is terminated, and the patient undergoes the process for titration of NIV during night time (TNT).

### TNT: titration of NIV during night time

The process for TNT is the last process of the workflow with three possible results (ref. Figure 1): If an adequate ventilation is achieved in terms of blood gas values, oxygenation (SpO_2_) and patient satisfaction, the patient is discharged (option 1). If the patient is not satisfied with the ventilation, blood gases or oxygenation are inadequate, adjustments to the ventilator and/or oxygen flow are made, and the patient undergoes the process for TNT again (option b). In addition, if the inspiratory pressure has to be adjusted, both processes TDT and TNT will be repeated (option 3).

For the process of TNT, the patient stays overnight, and ventilation is evaluated by blood gas analyses – once before the night without NIV and twice during the night with NIV. Values are fed to the process before and after the night time ventilation.

For blood gas analyses, the same safety rules as described for the process for TDT are applied by the algorithm. If SpO_2_ and pH values are within acceptable ranges, the questionnaire is applied and adjustments of the ventilator settings are recommended as delineated in Figure 2(b) and (c).

Since blood gas analysis during the night might influence patients sleep quality, other techniques like transcutaneous PCO_2_ (PtcCO_2_) measurements might be used during night time.^8^

1. Subsequently, the process of TNT ends with one of the three options delineated above.

## Conclusion

The DIGIVENT project established digital treatment algorithms and implemented a decision- and workflow-support system for NIV whose validation in a clinical cohort is planned.
